# The industrial solvent 1,4-dioxane causes hyperalgesia by targeting capsaicin receptor TRPV1

**DOI:** 10.1186/s12915-021-01211-0

**Published:** 2022-01-07

**Authors:** Xiaoyi Mo, Qiang Liu, Luna Gao, Chang Xie, Xin Wei, Peiyuan Pang, Quan Tian, Yue Gao, Youjing Zhang, Yuanyuan Wang, Tianchen Xiong, Bo Zhong, Dongdong Li, Jing Yao

**Affiliations:** 1 State Key Laboratory of Virology, College of Life Sciences, Department of Anesthesiology, Zhongnan Hospital of Wuhan University, Frontier Science Center for Immunology and Metabolism, Wuhan University, Wuhan, 430072 Hubei China; 2 Institute of Biology Paris Seine, Neuroscience Paris Seine, Sorbonne Université, CNRS UMR8246, INSERM U1130, UPMC UM119, 75005 Paris, France

**Keywords:** TRPV1, Hyperalgesia, 1,4-Dioxane, Nociception, Inflammatory pain

## Abstract

**Background:**

The synthetic chemical 1,4-dioxane is used as industrial solvent, food, and care product additive. 1,4-Dioxane has been noted to influence the nervous system in long-term animal experiments and in humans, but the molecular mechanisms underlying its effects on animals were not previously known.

**Results:**

Here, we report that 1,4-dioxane potentiates the capsaicin-sensitive transient receptor potential (TRP) channel TRPV1, thereby causing hyperalgesia in mouse model. This effect was abolished by CRISPR/Cas9-mediated genetic deletion of TRPV1 in sensory neurons, but enhanced under inflammatory conditions. 1,4-Dioxane lowered the temperature threshold for TRPV1 thermal activation and potentiated the channel sensitivity to agonistic stimuli. 1,3-dioxane and tetrahydrofuran which are structurally related to 1,4-dioxane also potentiated TRPV1 activation. The residue M572 in the S4-S5 linker region of TRPV1 was found to be crucial for direct activation of the channel by 1,4-dioxane and its analogs. A single residue mutation M572V abrogated the 1,4-dioxane-evoked currents while largely preserving the capsaicin responses. Our results further demonstrate that this residue exerts a gating effect through hydrophobic interactions and support the existence of discrete domains for multimodal gating of TRPV1 channel.

**Conclusions:**

Our results suggest TRPV1 is a co-receptor for 1,4-dioxane and that this accounts for its ability to dysregulate body nociceptive sensation.

**Supplementary Information:**

The online version contains supplementary material available at 10.1186/s12915-021-01211-0.

## Background

1,4-Dioxane (C_4_H_8_O_2_) is a heterocycliccompound widely used as an organic solvent or stabilizing agent in industries. It is found in some home cleaning products such as shampoo, mouthwash, and toothpaste. Its derivatives (e.g., 1,4-dioxane formaldehyde) are also used for cosmeceutical products to intensify their performance, quality, and life span [1, 2]. 1,4-Dioxane has been considered as a carcinogen based on long-term animal experiments [3]. In rats chronically exposed to 1,4-dioxane, damages were observed in the central nervous system, liver, and kidney [4, 5]. Recurrent influence was also observed for humans, ranging from a mild hypersensitivity to life-threatening anaphylaxis or lethal intoxication [6–8]. 1,4-Dioxane also acutely influences body sensory pathways, reflected by the irritation of the eyes, nose, throat, and lungs in humans [5, 9, 10]. Prolonged exposure thus harms the upper respiratory passages, symptomized by coughing and stomach pain. However, the in vivo molecular target of 1,4-dioxane and the action mechanism remain to be understood.

Primary sensory neurons in dorsal root, trigeminal, and nodose ganglia initiate pain in response to noxious chemical, mechanical, or thermal stimuli [11]. Transient receptor potential (TRP) channels are calcium-permeable and non-selective cation channels expressed by somatosensory neurons [12]. As molecular sensors for nociceptive stimulation, TRP channels prominently regulate the pathogenesis of both inflammatory and neuropathic pain [13]. The vanilloid receptor 1 (TRPV1) is predominantly expressed by sensory neurons [14]. It is a polymodal nociceptive receptor activated by capsaicin, heat above 42°C and other irritants such as protons and inflammatory mediators [15–17], whereby regulating pain perception [18, 19]. In addition, Kunkler and colleagues have demonstrated that daily exposures to environmental irritants like acrolein would induce functional sensitization of peripheral neural elements including TRPA1 and TRPV1 channels [20].

Here, using CRISPR-Cas9-engineered mouse model, we show that the nociceptive receptor TRPV1 is targeted by 1,4-dioxane in vivo, resulting in hyperalgesia behaviors. Combining electrophysiology, Ca^2+^ imaging, and molecular genetics, we demonstrate that 1,4-dioxane activates TRPV1 to cause Ca^2+^ influx in the dorsal root ganglia (DRG) and trigeminal ganglia (TG) sensory neurons. We further show that 1,4-dioxane potentiates the sensitivity of TRPV1 to inflammatory stimuli, while lowers its temperature threshold to thermal activation, hence providing a mechanistic basis for the observed hyperalgesia behavior. Our data suggest TRPV1 channel as a co-receptor for 1,4-dioxane, whereby it may dysregulate the nociceptive sensation.

## Results

### 1,4-Dioxane causes hyperalgesia via TRPV1 activation

**Fig. 1:**
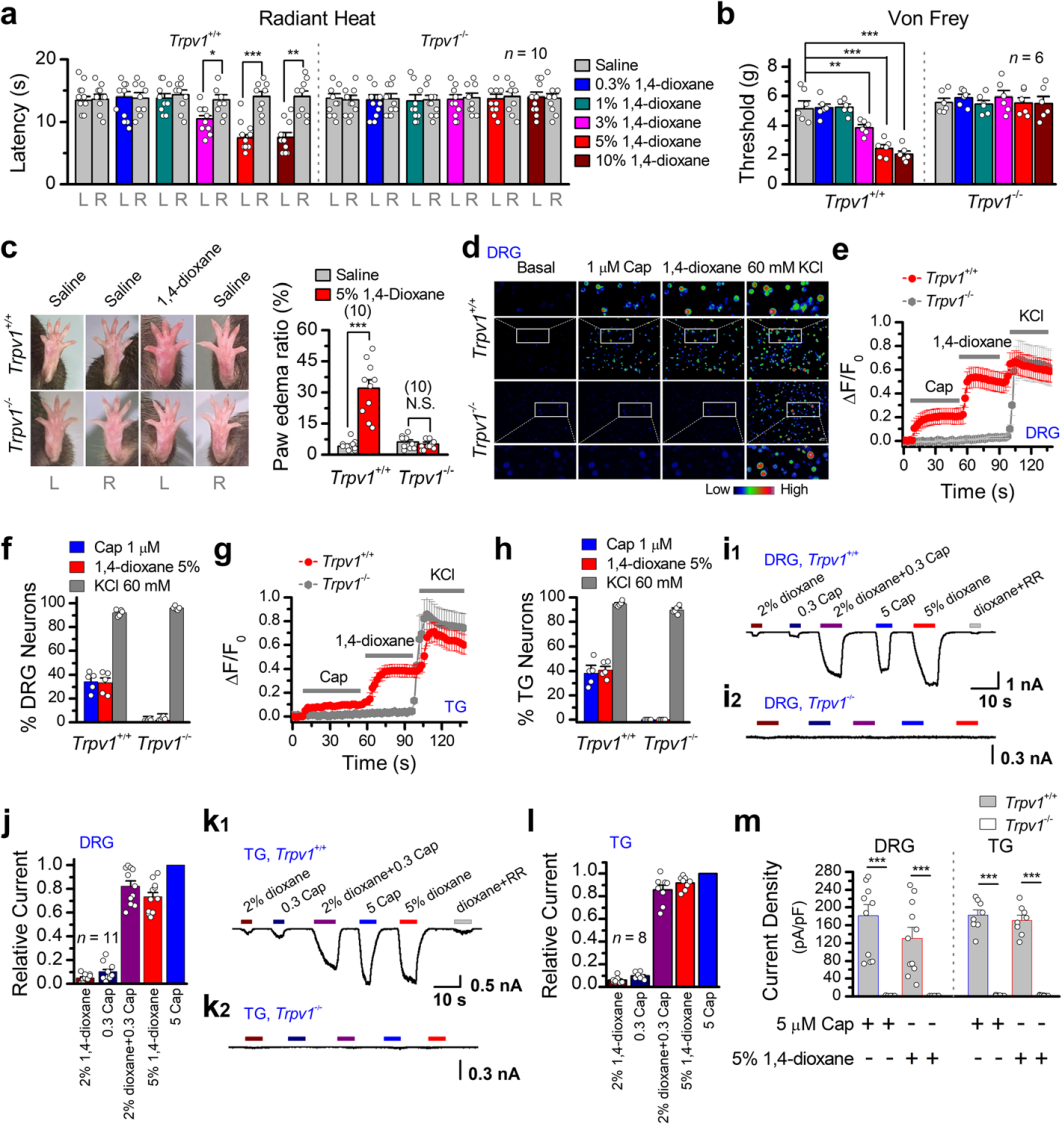
1,4-Dioxane causes mice hyperalgesia via TRPV1 activation. **a** The radiant heat test shows 1,4-dioxane-caused thermal hyperalgesia in WT mice but not in *Trpv1*^-/-^ mice. **b** Von Frey filament assay evaluating the effect of 1,4-dioxane on mechanical pain sensing. **c** Intraplantar injections of 10 μl of 5% 1,4-dioxane significantly increased paw volume compared with that injected with saline controls. The paw edema ratio is the percentage increase of paw volume induced by 1,4-dioxane in *Trpv1*^+/+^ mice. The effects of 1,4-dioxane were abolished in *Trpv1*^-/-^ mice. The representative images of the dioxane-injected paw edema are shown on the left. **d** Responses of dorsal root ganglia (DRG) neurons acutely isolated from *Trpv1*^+/+^ or *Trpv1*^-/-^ mice were consecutively challenged with 1 μM capsaicin (Cap), 5% 1,4-dioxane, and 60 mM KCl, as indicated. Channel activation was assessed by calcium imaging in cells loaded with the fluorescent Ca^2+^ indicator Fluo-4, AM. The colored bar indicates relative calcium levels. **e** Averaged responses of DRG neurons dissociated from *Trpv1*^+/+^ (gray, *n* = 61) or *Trpv1*^-/-^ (red, *n* = 45) mice to capsaicin, 1,4-dioxane, and KCl. Fluo-4 epifluorescence changes were computed as (Fi–F0)/F0, where Fi represented fluorescence intensity at any frame and F0 was the baseline fluorescence calculated from the averaged fluorescence of the first 10 frames. **f** Percentage of DRG neurons responding to capsaicin (1 μM), 1,4-dioxane (5%), or high KCl (60 mM) in neurons isolated from *Trpv1*^+/+^ or *Trpv1*^-/-^ mice. **g** Trigeminal ganglia (TG) neurons from *Trpv1*^+/+^ or *Trpv1*^-/-^ mice were challenged with 1,4-dioxane (5%) followed by capsaicin (1 μM), then high KCl (60 mM). Quantification of responses were assessed by calcium imaging (*n* ≥ 30 cells per trace). **h** Percentage of TG neurons responding to capsaicin, 1,4-dioxane, or high KCl in neurons isolated from *Trpv1*^+/+^ or *Trpv1*^-/-^ mice. **i** Typical response of DRG neurons cultured from wild-type (**i1**) and *Trpv1*-deficient (**i2**) mice to 1,4-dioxane and capsaicin. **j** Quantification of peak currents for recordings in (**i1**). **k–l** Similar recordings and statistical results in TG neurons. **m** Quantification of responses to 1,4-dioxane (5%) and capsaicin (5 μM) in both DRG and TG neurons cultured from wild-type or *Trpv1*^-/-^ mice (*n* ≥ 8). Responses to 1,4-dioxane were observed only in the capsaicin sensitive neurons

1,4-Dioxane penetrates the skin and irritates multiple tissues in humans [9, 10]. To explore its effect on pain transduction, we used the Radiant Heat and Von Frey assay to respectively evaluate thermal and mechanical sensation in mouse model. Varying concentrations of 1,4-dioxane as indicated or normal saline (10 μl) were injected into the mouse left hind paw while using the right hind paw as control (10 μl saline only). After 30 min, we counted the withdrawal latency of each hind paw in response to radiant heat as nocifensive reaction (i.e., licking foot). The paw withdrawal latency was significantly reduced along with increased concentrations of 1,4-dioxane (e.g., 7.6 ± 0.7 s for administration of 10% 1,4-dioxane vs. 14.1 ± 0.8 s of the control paw, *p* < 0.01) (Fig. 1a), reflecting an enhanced sensitivity to high temperature stimulus. In Von Frey assessment, 1,4-dioxane paw injection also decreased the threshold for mechanical pain sensing (e.g., painful weight = 2.1 ± 0.2 g for administration of 10% 1,4-dioxane vs. 5.4 ± 0.4 g of the control paw, *p* < 0.001) (Fig. 1b). Therefore, 1,4-dioxane causes both thermal and mechanical hyperalgesia.

Sensory transduction in mammalian animals is importantly mediated by ion channels, among which the TRP channels expressed by the peripheral sensory neurons play an essential role. We then investigated the effects of 1,4-dioxane on nociceptive TRP ion channels known to be expressed in DRG neurons. TRPV1, TRPA1, TRPV2, TRPM8, TRPV3, and TRPM3 channels were individually expressed in HEK 293 cells and consecutively stimulated by their own agonist and 1,4-dioxane. As illustrated in Fig. S1, among the tested TRP channels, only TRPV1 showed response currents in response to 3% or 5% 1,4-dioxane, indicating its direct effect on the TRPV1 channel.

Previous studies have shown that TRPV1 is closely related to mechanical and thermal hypersensitivity [21, 22], thus suggesting that 1,4-dioxane may cause thermal and mechanical hyperalgesia by activating TRPV1. To explore the involvement of TRPV1 in 1,4-dioxane-caused hyperalgesia, we used a mouse model where the *Trpv1* gene was deleted by CRISPR/Cas9-mediated genome editing (Fig. S2a). Consistent with previous studies, knockout of TRPV1 had no obvious effect on the development of mice [18], nor on their general appearance, gross anatomy, body weight, locomotion, or overt behavior. However, the 1,4-dioxane-caused hyperalgesia was completely lost in *Trpv1*^-/-^ mice (Fig. 1a, b), indicating that TRPV1 channel mediates the effect of 1,4-dioxane.

Previous studies have shown that the direct activation of TRPV1 resulted in the paw edema in mice [23–25]. We next asked whether 1,4-dioxane can also elicit paw edema in vivo. As illustrated in Fig. 1c, we found that intraplantar administration of 1,4-dioxane (5%, 10 μl) produced a significant increase in paw volume from 4 ± 1 to 32 ± 4% in the hind paws of wild-type mice. This scenario was abolished in *Trpv1*^*-/-*^ mice, confirming that the effect of 1,4-dioxane is TRPV1-dependent.

As TRPV1 is highly permeable to Ca^2+^, we examined its activation by 1,4-dioxane with calcium imaging. We observed that 1,4-dioxane evoked robust Ca^2+^ increases in acutely isolated TG and DRG neurons of wild-type (WT) mice, but not in neurons prepared from *Trpv1* KO mice (Fig. 1d–h). High KCl (60 mM) was subsequently applied to ascertain neuronal viability in the end of each experiment. Patch-clamp recordings further revealed that 1,4-dioxane (2%) potentiated the capsaicin-activated TRPV1 current in DRG and TG sensory neurons and was also able to directly trigger TRPV1 currents in a concentration-dependent manner (Fig. 1i–m). As expected, the effect of 1,4-dioxane was fully suppressed in neurons of *Trpv1* KO mice. Together, 1,4-dioxane-caused hyperalgesia is mediated by the enhanced activity of TRPV1 channel.

### Gating and modulation of TRPV1 by 1,4-dioxane

**Fig. 2:**
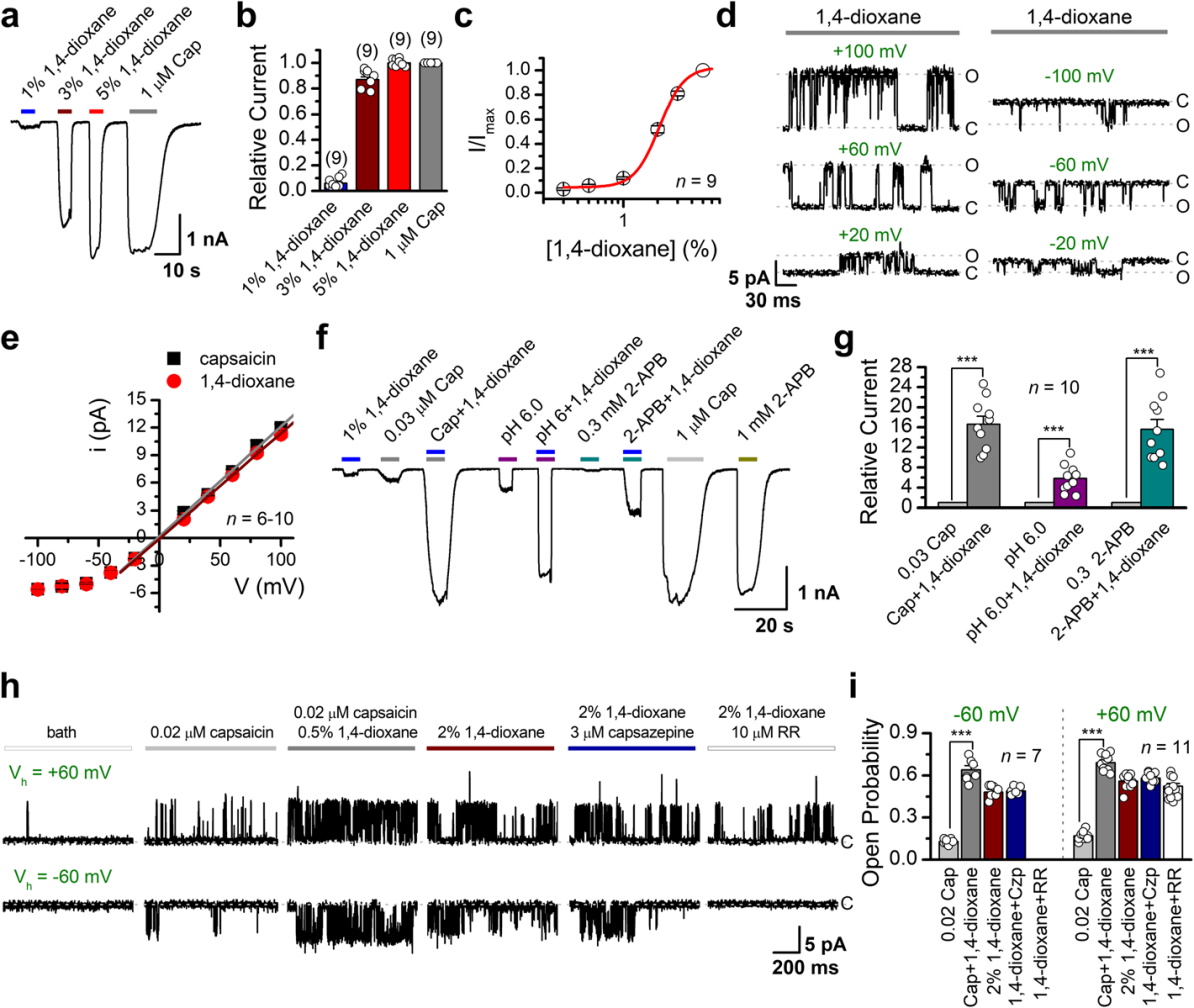
Gating and modulation of TRPV1 channels by 1,4-dioxane. **a** Representative whole-cell recordings from TRPV1-expressing HEK293 cell. The cell was exposed to varied concentrations of 1,4-dioxane or capsaicin (Cap, 1 μM), as indicated. Holding potential was −60 mV. **b** Summary of relative currents elicited by 1,4-dioxane or capsaicin. Currents were normalized to that evoked by 1 μM capsaicin. Numbers of cells are indicated in parentheses. **c** Dose-response curve of 1,4-dioxane. Fitting by Hill’s equation gave an EC_50_ = 2.1 ± 0.03% and n_H_ = 3.1 ± 0.22 (*n* = 9). **d** Representative single-channel currents recorded from an outside-out patch of TRPV1-expressing HEK293 cells. Currents were evoked by 1,4-dioxane at different voltages ranging from −100 to +100 mV and were low-pass filtered at 2 kHz. **e** Unitary current-voltage relationships activated by 1,4-dioxane (red) or capsaicin (black), showing no significant difference. **f** Potentiation effects of 1,4-dioxane. Currents were evoked by capsaicin, 2-APB, acidification (pH 6.0) or 1,4-dioxane as indicated. The presence of 1,4-dioxane potentiated the responses of each stimulation. Holding potential V_h_ = −60 mV. **g** Comparison of the averaged response evoked by different agonists with or without 1% 1,4-dioxane. The labels over the bars indicate the number of recordings. **h** Potentiation by 1,4-dioxane of single-channel activity in outside-out patches excised from TRPV1-expressing HEK293 cells. Capsaicin, 1,4-dioxane, capsazepine, or RR were applied consecutively to the same patches as indicated. The holding potentials were +60 mV (*upper*) and −60 mV (*lower*), respectively. Co-application of 1,4-dioxane (0.5%) with 0.02 μM capsaicin resulted in more single-channel openings. **i** Average changes in TRPV1 open probability. The addition of 1,4-dioxane remarkably increased single-channel open probability. Inclusion of RR but not capsazepine reduced the channel openings (V_h_ = −60 mV, *n* = 7–11 cells per condition)

To characterize 1,4-dioxane potentiation of TRPV1 channel, we expressed it in HEK293 cells and used whole-cell voltage-clamp recording to analyze the electrophysiological responses**.** As shown in Fig. 2a–c, 1, 4-dioxane dose-dependently activated TRPV1 channel with an EC_50_ = 2.1 ± 0.03% concentration. Single-channel recordings were acquired from the outside-out membrane patches of TRPV1-expressing HEK293 cells (Fig. 2d), perfused with 1,4-dioxane under various voltage clamps ranging from −100 to +100 mV. Comparing the i-V curve between 1,4-dioxane and capsaicin activation, we observed a similar rectified shape with a relatively linear increase at depolarizing voltages and a sublinear dependence at hyperpolarizing potentials (Fig. 2e).

TRPV1 is a multimodal channel and can be activated by various stimuli such as voltage, heat, protons, capsaicin, and the pharmacological compound 2-aminoethoxydiphenyl borate (2-APB) as well as a variety of endogenous factors. Notably, one type of activation can be potentiated by another. We assessed the impact of 1,4-dioxane on TRPV1 activation by other stimulations. As shown in Fig. 2f, g, 1, 4-dioxane indeed potentiated TRPV1 currents evoked by capsaicin, acidification (pH 6.0), and 2-APB. A scenario was further confirmed by single-channel recordings showing that the presence of 1,4-dioxane increased TRPV1 channel open probability (Fig. 2h, i). There was no significant difference for the amplitude of single-channel currents elicited by different conditions (Fig. S3). The higher opening of the channel was associated with stronger noise, which tended to be more profound at hyperpolarized potentials. Notably, the single-channel currents evoked by 1,4-dioxane were fully blocked by TRPV1 blocker ruthenium red (RR, 10 μM, −60 mV, Fig. 2h) but not capsazepine (Czp, Fig. 2i), the selective inhibitor for capsaicin stimulation. Notably, RR did not block TRPV1 currents evoked by either 1,4-dioxane or capsaicin at +60 mV (Fig. S4). These data indicate that 1,4-dioxane potentiates TRPV1 activity at the single-channel level.

### 1,4-Dioxane lowers temperature threshold for TRPV1 activation

**Fig. 3:**
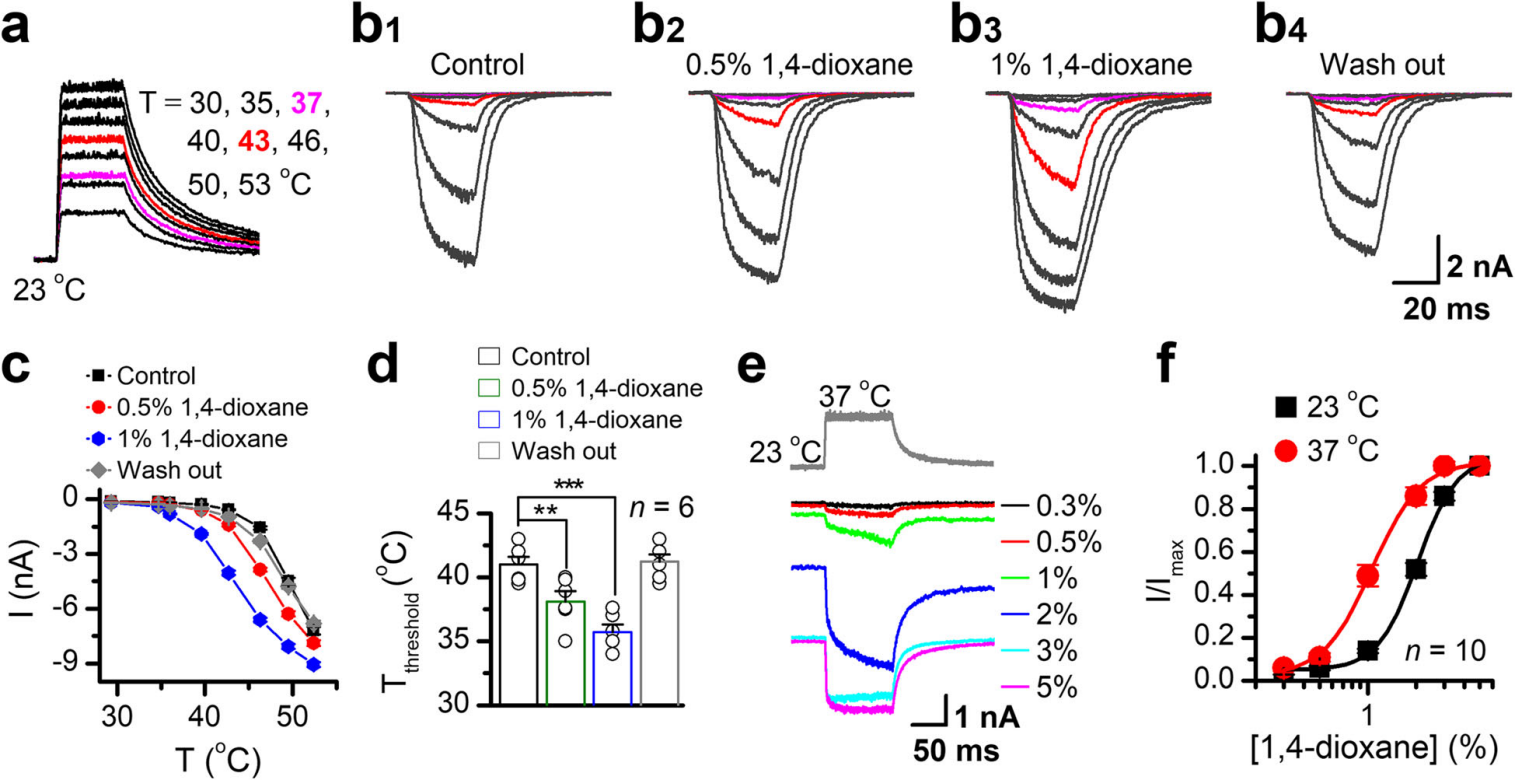
1,4-Dioxane lowers the temperature threshold of TRPV1 thermal activation. **a** Ultrafast temperature jumps from 30 to 53°C generated by infrared laser irradiation. Temperature pulses at 37°C and 43°C are shown in purple and red, respectively. **b** TRPV1 thermal responses before and after 1,4-dioxane treatment: **b1**, responses at control; **b2** and **b3**, responses to same temperature in the presence of 0.5% and 1% 1,4-dioxane, respectively; and **b4**, responses after washout. Holding potential was −60 mV. **c** Comparison of current-temperature relationships. Temperature response curves were measured from the maximal current at the end of each temperature step. Shown are representatives of six independent experiments with similar results. **d** Summary effects of 1,4-dioxane on the temperature threshold (*T*_threshold_) changes, *T*_threshold_ = 41.0 ± 0.6°C and 41.3 ± 0.5°C for control (*n* = 6) and washout (*n* = 6), *T*_threshold_ = 38.1 ± 0.8°C and 35.7 ± 0.6°C for the treatment with 0.5% (*n* = 6), and 1% (*n* = 6) 1,4-dioxane, respectively. **e** Whole-cell TRPV1 currents evoked by variable 1,4-dioxane concentrations at 23°C and 37°C. Heat-evoked current traces were recorded in whole-cell configuration from TRPV1-expressing HEK 293 cells held at −60 mV. The temperature pulse (37°C) is shown in gray. **f** Dose dependence of 1,4-dioxane for data in **e**. The solid lines represent fit to Hill’s equation with EC_50_ = 2.03 ± 0.05% and nH = 3.5 ± 0.39 at 23°C (*n* = 10), and EC_50_ = 1.04 ± 0.02% and nH = 2.9 ± 0.5 at 37°C (*n* = 10)

TRPV1 is a thermal sensor responding to noxious temperature for pain initiation. We tested whether 1,4-dioxane affects the temperature threshold of TRPV1. With an infrared laser system, ultrafast temperature sweeps (30 to 53°C) were generated surrounding TRPV1-expressing HEK293 cells (Fig. 3a). As previously reported [26], TRPV1 appeared to be activated at a temperature threshold of ~43^o^C (Fig. 3b_1_). The presence of 1,4-dioxane dose-dependently strengthened the thermal sensitivity of TRPV1, with the temperature threshold dropping down to 37°C (Fig. 3b–d). We then asked if temperature also affects 1,4-dioxane activation of TRPV1. As shown in Fig. 3e, TRPV1 currents were evoked by various concentrations of 1,4-dioxane, during which a temperature jump from 23 to 37°C was introduced. As the temperature increased, the activation effect of 1,4-dioxane became much stronger. The EC_50_ of 1,4-dioxane on TRPV1 activation was shifted to 1.04 ± 0.02% from 2.03 ± 0.05% when temperature was increased from 23 to 37°C (Fig. 3f). The interactive facilitation of 1,4-dioxane with TRPV1 thermal activation provides a mechanistic basis for 1,4-dioxane-caused hyperalgesia.

### Inflammation-related factors enhance 1,4-dioxane activation of TRPV1

**Fig. 4:**
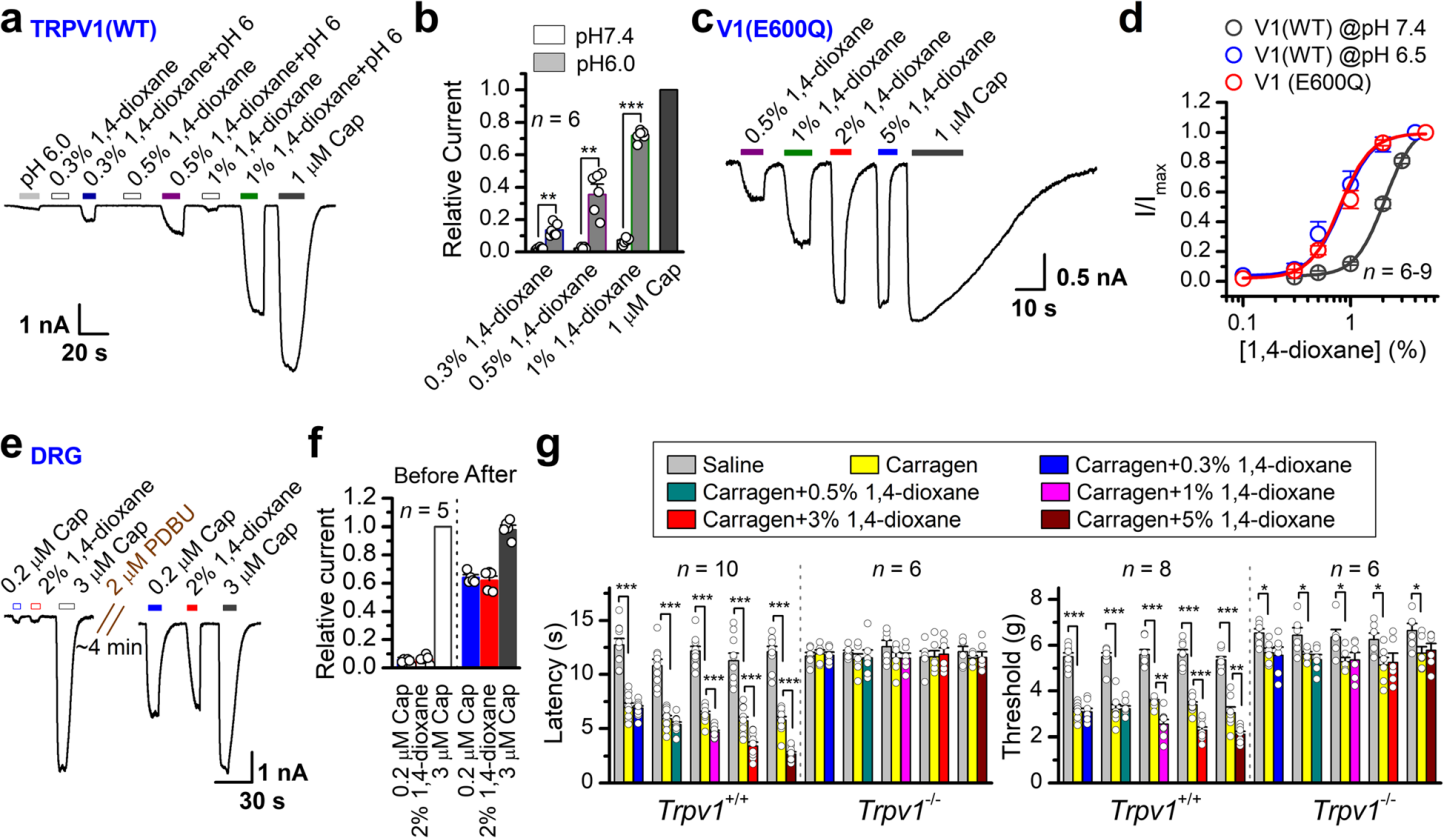
Activation of TRPV1 under inflammation-related conditions. **a** Effects of acid on the 1,4-dioxane-induced response in TRPV1-expressing HEK293 cells. Representative current traces evoked by varied concentrations of 1,4-dioxane in combination with the neutral pH (7.4) and then switched to pH 6.0. Acid strongly potentiated 1,4-dioxane responses at 0.3%, 0.5%, and 1% concentration. **b** The average plot compares the 1,4-dioxane response in pH 7.4 and 6.0 conditions. Currents were normalized to the responses evoked by 1 μM capsaicin. **c** A representative whole-cell recording of the TRPV1 E600Q mutant. The cell was exposed to different concentrations of 1,4-dioxane in neutral condition (pH 7.4). **d** Average plot of peak currents. The constitutively acidified TRPV1 E600Q mutant potentiated 1,4-dioxane-evoked current responses. **e** Potentiation of 1,4-dioxane (2%) responses by PDBu treatment in primary cultured DRG neurons. The phosphorylation of the channel induced by PDBu (2 μM) was verified with two capsaicin concentrations, 0.2 and 1 μM, applied before and after treatment. After phosphorylation, 1,4-dioxane (2%) or capsaicin (0.2 μM) elicited larger responses. The pipette solution also contained 2 mM MgATP. **f** Relative changes of the 1,4-dioxane- and capsaicin-evoked responses before and after phosphorylation. All recordings were made at a membrane potential of −60 mV. **g** The PWL of hind paws to radiant heat were measured for *Trpv1*^*+/+*^ and *Trpv1*^*-/-*^ mice under carrageenan-induced inflammation conditions (*left*). Von Frey filament assay evaluating the effect of 1,4-dioxane on mechanical pain sensing under carrageenan-induced inflammation conditions (*right*)

Tissue acidosis is a feature of pain-caused local inflammation [27, 28] and contributes to nociceptive hypersensitivity [29]. We tested whether the effect of 1,4-dioxane is enhanced under this inflammatory state. To mimic local acidification, we examined the effect of low pH on 1,4-dioxane activation. In whole-cell configuration, while pH 6.0 alone induced a small TRPV1 current, it significantly increased the TRPV1 currents evoked by 1,4-dioxane (Fig. 4a–b). Within TRPV1 channel, the protonation of glutamic residue (E600) regulates acid potentiation [30]. Neutralization of the residue E600 by glutamine (Q) yields a constitutively protonated TRPV1 mutant TRPV1(E600Q). 1,4-Dioxane was observed to evoke much larger currents (Fig. 4c) than those observed with WT channel in neutral conditions (Fig. 4a–b). Furthermore, lowering pH resulted in a similar leftward shift of the concentration-response curve to 1,4-dioxane as the observed for TRPV1(E600Q) mutant; EC_50_ was reduced from 2.1 ± 0.03% at pH 7.4 to 0.83 ± 0.03% at pH 6.5 for wild-type TRPV1 and the EC_50_ value for E600Q was determined as 0.79 ± 0.02% at pH 7.4 (Fig. 4d). The steepness of the curve remains largely unchanged (wild-type: n_H_ = 3.17 ± 0.22 at pH7.4 and n_H_ = 3.05 ± 0.55 at pH 6.5; E600Q: n_H_ = 3.08 ± 0.24 at pH 7.4). Hence, acidification state potentiates 1,4-dioxane activation of TRPV1.

Phosphorylation modification represents another inflammatory response, in association with acidic microenvironment [28]. We applied phorbol 12, 13-dibutyrate (PDBu, 2 μM, ~5 min), an activator of protein kinase C, to phosphorylate TRPV1-expressing DRG neurons [31]. This treatment caused a ~10-fold increase in the TRPV1 current evoked by 2% 1,4-dioxane (Fig. 4e), an effect comparably seen for capsaicin-evoked current (Fig. 4f).

To explore the effect of 1,4-dioxane on pain transduction under inflammation condition, we injected carrageenan (20 μL of 2% (w/v) in normal saline) into the hindpaws of mice to induce a state of local inflammation [19, 32]. In half an hour, both the withdrawal latency of the injected paws in response to radiant heat and the threshold for mechanical pain sensing decreased dramatically to about 50% of the baseline level in *Trpv1*^*+/+*^ mice (Fig. 4g). However, carrageenan-induced thermosensation was completely lost and carrageenan-induced mechanosensation was partially lost in *Trpv1*^*-/-*^ mice (Fig. 4g). Thereafter, varying concentrations of 1,4-dioxane were injected into the same hind paws post carrageenan injection. As compared to non-inflammatory conditions (Fig. 1a–b), lower concentration of 1,4-dioxane could significantly reduce the paw withdrawal latency to heat and the mechanical pain sensitivity in mice under an inflammatory state (Fig. 4g). Again, the 1,4-dioxane-caused hyperalgesia under inflammation condition was completely lost in *Trpv1*^*-/-*^ mice.

These data, together, show that the activation of TRPV1 by 1,4-dioxane is enhanced in inflammatory conditions.

### Activation of TRPV1 by 1,4-dioxane and its analogs is mediated by M572 residue

**Fig. 5:**
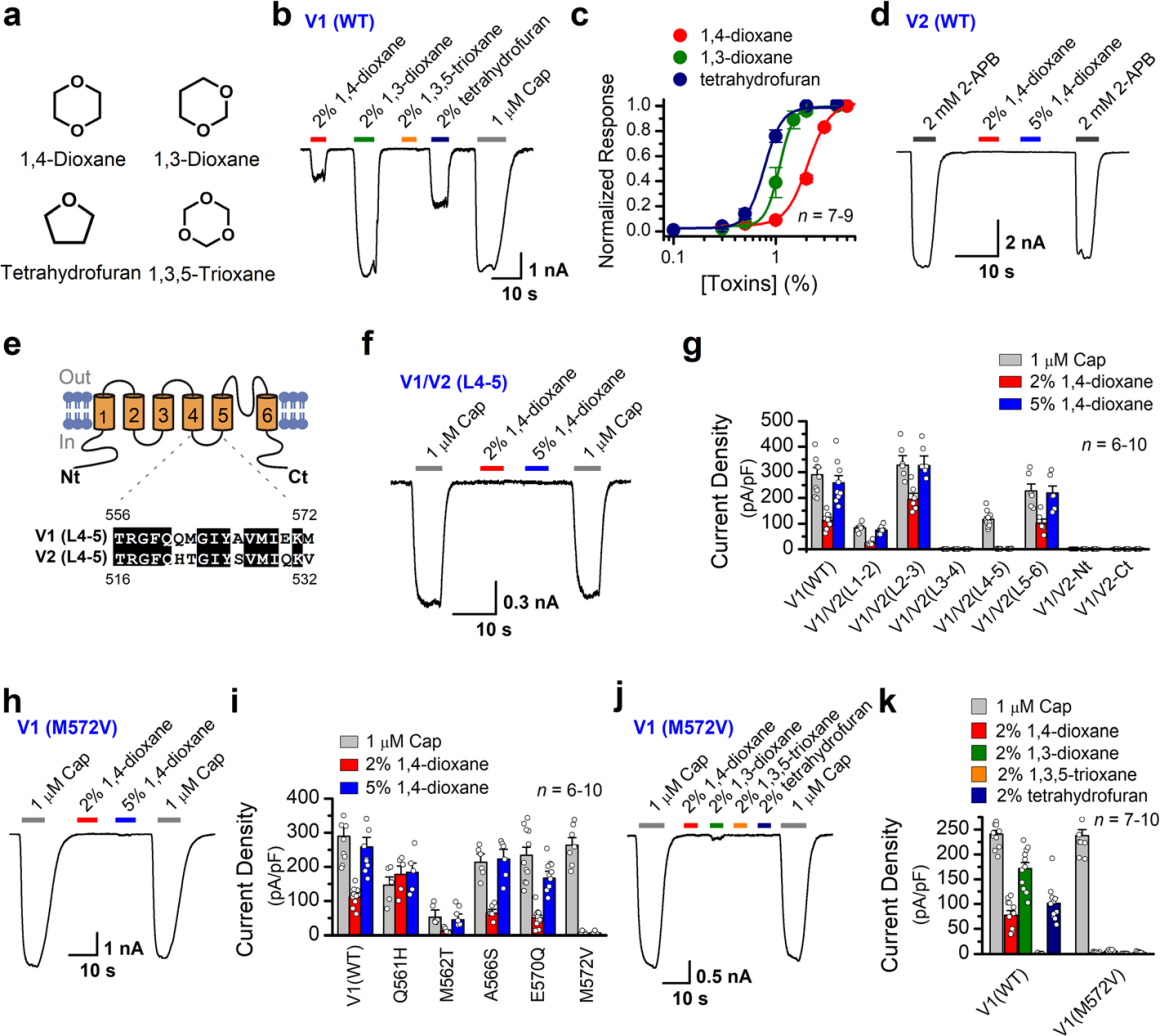
M572 residue in TRPV1 S4-S5 linker region mediates the effect of 1,4-dioxane and its analogs. **a** Structures of 1,3-dioxane, tetrahydrofuran, and 1,3,5-trioxane compared with that of 1,4-dioxane. **b** Activation of TRPV1 by 1,4-dioxane analogs. Representative trace of TRPV1 whole-cell currents from TRPV1-expressing HEK293 cells evoked by 1,4-dioxane (2%), 1,3-dioxane (2%), 1,3,5-trioxane (2%), tetrahydrofuran (THF, 2%), and capsaicin (Cap, 1 μM) at −60 mV. **c** Concentration-response curves of whole-cell TRPV1 currents evoked by 1,3-dioxane and tetrahydrofuran, respectively. **d** Representative whole-cell recording from TRPV2-expressing HEK293 cell showing its responses to 2-APB and insensitivity to 1,4-dioxane. **e** *Top*, putative membrane topology of a single TRPV1 subunit. *Bottom*, amino acid alignment of the linker of S4-S5 between TRPV1 and TRPV2, with the identical residues shaded in black. **f** Representative whole-cell recordings showing that 1,4-dioxane failed to activate V1/V2(L4-5) chimeric channel, which is a TRPV1 mutant with the linker for S4-S5 swapped by the cognate segment of rat TRPV2; however, the response to capsaicin (Cap) was retained. **g** Summary data of current densities at −60 mV evoked by capsaicin and 1,4-dioxane obtained by whole-cell recordings. Rat TRPV1 mutants with the N terminus (Nt), linker S2-S3 (L1-2), linker S2-S3 (L2-3), linker S2-S3 (L3-4), linker S4-S5 (L4-5), linker S5-S6 (L5-6), and the C terminus (Ct) swapped by the cognate segments of rat TRPV2, respectively. **h** Representative whole-cell recordings showing that mutant channel V1(M572V) lost sensitivity to 1,4-dioxane, though the response to capsaicin was retained. **i** Average responses of the mutant channels evoked by different stimuli as indicated. Residues in the linker S4-S5 of TRPV1 were individually mutated to the corresponding residues of TRPV2. Whole-cell currents were recorded from transiently transfected HEK293 cells at −60 mV, and current densities were determined by dividing the peak current amplitudes by membrane capacitance*.* **j** Whole-cell recordings were made at −60 mV from HEK293 cells showing that 1,4-dioxane (2%), 1,3-dioxane (2%), and tetrahydrofuran (2%) failed to activate V1(M572V), while the response to capsaicin (1 μM) was retained. **k** Summary data of current densities at −60 mV evoked by capsaicin (1 μM), 1,4-dioxane (2%), 1,3-dioxane (2%), and 1,3,5-trioxane (2%) and tetrahydrofuran (2%) obtained by whole-cell recordings

Structurally related to 1,4-dioxane, 1,3-dioxane, 1,3,5-trioxane, and tetrahydrofuran are also widely used solvents [33] (Fig. 5a). 2% 1,3-dioxane or 2% tetrahydrofuran efficiently activated TRPV1, but no measurable current was evoked by 2% 1,3,5-trioxane (Fig. 5b). Fitting the dose-response curves with the Hill equation resulted in an EC_50_ = 1.08 ± 0.02% and n_H_ = 6.35 ± 0.84 for 1,3-dioxane (*n* = 7), and an EC_50_ = 0.77 ± 0.03%, and n_H_ = 5.05 ± 0.57 for tetrahydrofuran (*n* = 8; Fig. 5c).

We next sought to define the molecular determinant(s) of TRPV1 activation by 1,4-dioxane and its analogs. We noticed that rat TRPV2 though sharing ~70% amino acid similarity with rat TRPV1 [34] is insensitive to 1,4-dioxane (Fig. 5d). We therefore constructed a series of TRPV1/V2 chimeras with the segments of TRPV1 being replaced by the cognate region of TRPV2 (Fig. 5e). The resultant chimeras were designated as TRPV1/V2(Nt), TRPV1/V2(L1-2), TRPV1/V2(L2-3), TRPV1/V2(L3-4), TRPV1/V2(L4-5), TRPV1/V2(L5-6), and TRPV1/V2(Ct). We tested 1,4-dioxane activation following transient expression of the chimerical channels in HEK293 cells (Fig. 5)f–g. Three chimeras, TRPV1/V2(Nt), TRPV1/V2(L3-4), and TRPV1/V2 (Ct), lost their responses to both 1 μM Cap and 2 or 5% 1,4-dioxane stimulation. These stimulations evoked current responses in TRPV1/V2(L2-3) and TRPV1/V2(L5-6) mutants, yet no significant difference in current density was observed as compared with the responses obtained with wild-type TRPV1(WT). For TRPV1/V2(L1-2) chimera, although the responses to capsaicin or 1,4-dioxane were reduced, the activities evoked by different stimuli still remained. In contrast, the TRPV1/V2(L4-5) chimera lost full responsiveness to 1,4-dioxane, without affecting capsaicin-evoked current (Fig. 5f–g). Therefore, the S4-S5 linker region of TRPV1 appears critical for the effect of 1,4-dioxane. We screened therein five different residues between TRPV1 and TRPV2 based on the sequence alignment (Fig. 5e) and constructed TRPV1 mutants by replacing the different residues individually with TRPV2 counterparts. By whole-cell recording, we found that the sensitivity of TRPV1 to 1,4-dioxane was selectively abolished in M572V mutant (Fig. 5h–i), thus indicating a pivotal role for M572 residue in 1,4-dioxane-mediated TRPV1 activation. Indeed, reconstitution of TRPV1(M572V) in DRG neurons of *Trpv1*^-/-^ mice restored the response to capsaicin but not to 1,4-dioxane (Fig. S5). We also observed that 1,3-dioxane or tetrahydrofuran failed to activate TRPV1(M572V) mutant (Fig. 5j, k), whereby corroborating the essential requirement of the M572 residue for TRPV1 activation as observed with 1,4-dioxane.

### Side-chain property of M572

**Fig. 6:**
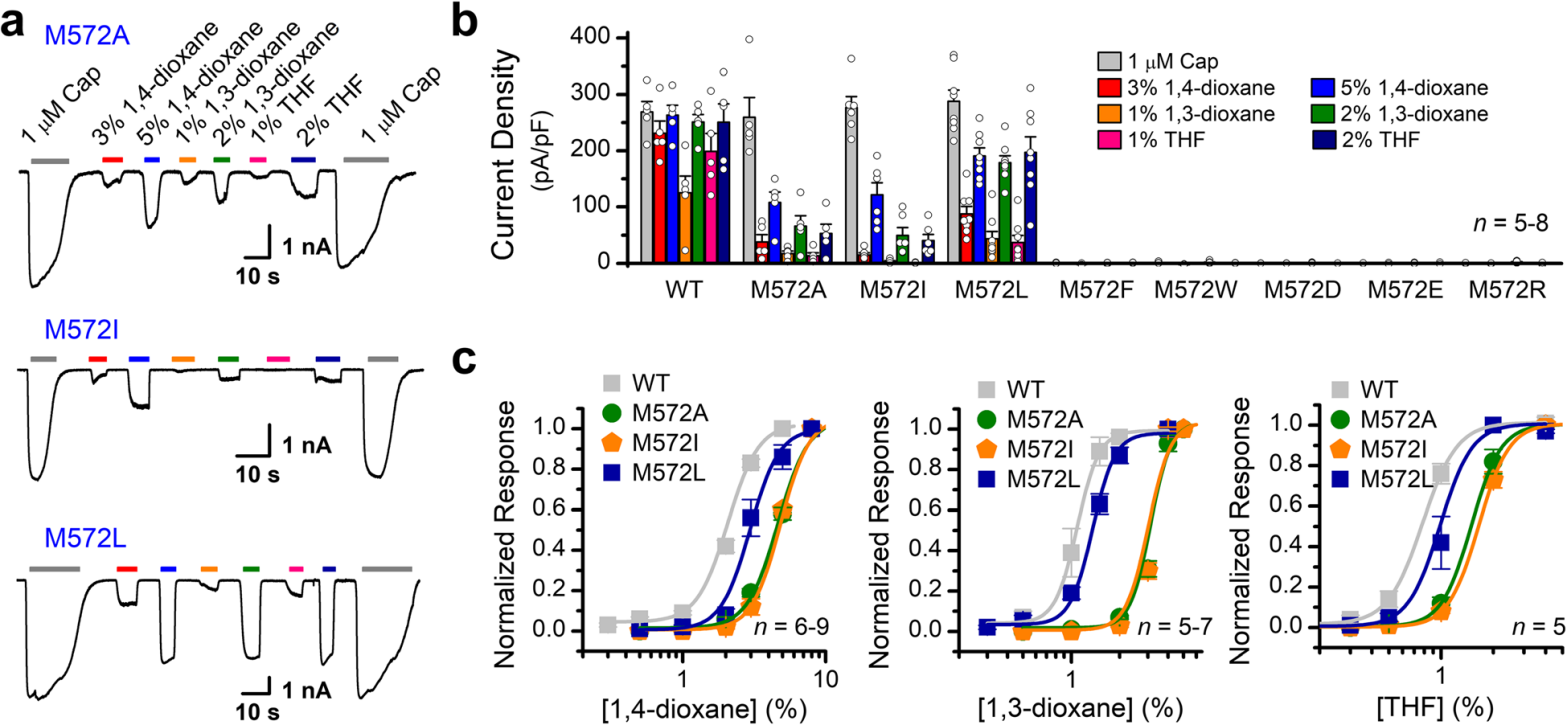
Side-chain property at position 572. **a** Representative whole-cell recordings from HEK293 cells expressed TRPV1(M572A), TRPV1(M572I), or TRPV1(M572L) evoked by 1,4-dioxane (3% and 5%), 1,3-dioxane (1% and 2%), tetrahydrofuran (THF, 1% and 2%), and capsaicin (Cap, 1 μM). Holding potential was −60 mV. **b** Averaged responses of the mutant TRPV1 channels evoked by different stimuli as indicated. Current densities were determined by dividing the peak current amplitudes by membrane capacitance. **c** Concentration-response curves of mutant TRPV1 channels. Solid lines are fits to Hill’s equation. For 1,4-dioxane, EC_50_ = 4.64 ± 0.24%, and n_H_ = 4.03 ± 0.75 for V1(M572A) (*n* = 9); EC_50_ = 4.83 ± 0.24%, and n_H_ = 4.33 ± 0.90 for V1(M572I) (*n* = 6); EC_50_ = 2.96 ± 0.13%, and n_H_ = 4.26 ± 0.76 for V1(M572L) (*n* = 8). For 1,3-dioxane, EC_50_ = 3.11 ± 0.06 %, and n_H_ = 6.88 ± 0.96 for V1(M572A) (*n* = 6); EC_50_ = 3.02 ± 0.04%, and n_H_ = 6.22 ± 0.55 for V1(M572I) (*n* = 5); EC_50_ = 1.36 ± 0.05%, and n_H_ = 6.25 ± 1.02 for V1(M572L) (*n* = 7). For tetrahydrofuran, EC_50_ = 1.49 ± 0.02%, and n_H_ = 5.08 ± 0.15 for V1(M572A) (*n* = 5); EC_50_ = 1.65 ± 0.01%, and n_H_ = 4.97 ± 0.12 for V1(M572I) (*n* = 5); EC_50_ = 0.99 ± 0.01%, and n_H_ = 4.73 ± 0.35 for V1(M572L) (*n* = 5)

To understand what feature of M572 makes it play an important role in the 1,4-dioxane-evoked TRPV1 activation, in addition to the mutation M572V, we further constructed a series of new substitutions at this position including L, I, W, F, A, D, E, and R, which span both polarity and size. Among eight new mutants of TRPV1, the substitutions by A, L, and I caused a significant reduction in responses to 1,4-dioxane while retaining >95% of the wild-type capsaicin-evoked activity (Fig. 6a–b). The substitutions of M by F or W with an aromatic side chain, as well as the hydrophilic and charged residue R, E, or D, all resulted in nonfunctional channels (Fig. 6b). Mutations M572V, M572A, M572I, and M572L with different side-chain size showed varied sensitivity to 1,4-dioxane and its analogs. The dose-response curves were fitted with a Hill equation, and the corresponding EC_50_ values for each TRPV1 mutant were as follows: for 1,4-dioxane, EC_50_ = 4.64 ± 0.24% for V1(M572A), EC_50_ = 4.83 ± 0.24% for V1(M572I), EC_50_ = 2.96 ± 0.13% for V1(M572L); for 1,3-dioxane, EC_50_ = 3.11 ± 0.06% for V1(M572A), EC_50_ = 3.02 ± 0.04% for V1(M572I), and EC_50_ = 1.36 ± 0.05% for V1(M572L); for tetrahydrofuran, EC_50_ = 1.49 ± 0.02% for V1(M572A), EC_50_ = 1.65 ± 0.01% for V1(M572I), and EC_50_ = 0.99 ± 0.01% for V1(M572L) (Fig. 6c). Together, these results suggest that the hydrophobic side-chain of the residue M572 with proper steric hindrance is critical for TRPV1 activation evoked by 1,4-dioxane and its analogs.

## Discussion

As a trace contaminant, 1,4-dioxane is used as an organic solvent in the industry and could be found in some daily care products and food additives. Here, we show that 1,4-dioxane causes hyperalgesia by potentiating the nociceptive receptor TRPV1. Our data reveal that TRPV1 activation is acutely facilitated by 1,4-dioxane. Moreover, our results suggest that TRPV1 potentiation may occur in conditions exposing to high levels of 1,4-dioxane. As TRPV1 channel can be gradually sensitized, recurrent exposure to 1,4-dioxane may also disturb TRPV1-mediated sensory transduction. We also observed that 1,4-dioxane analogs 1,3-dioxane and tetrahydrofuran are also able to activate TRPV1. As 1,4-dioxane is widely used solvents in industry [33], for instance, up to 8% of 1,4-dioxane is used in the chlorinated solvent 1,1,1-trichloroethane [TCA] and even higher concentration used in certain cutting oils [35], our data suggest appropriate measures need be taken to avoid their potential interference with sensory transduction.

1,4-Dioxane caused hyperalgesia in vivo within minutes, compatible with its skin penetration property and consistent with its irritating effect on humans [5]. Such scenario differs from the chronic effect of 1,4-dioxane that underlies organ damage and tumorigenesis [7]. We attributed 1,4-dioxane-caused hyperalgeisa to its action on TRPV1 receptor as evidenced by CRISPR/Cas9 KO genetics, which is consistent with the wide distribution of TRPV1 in nociceptive sensory neurons and its role in nociception [36, 37]. While our current data demonstrate TRPV1 as a molecular target for 1,4-dioxane, it could also dysregualte other sensory pathways.

TRPV1 functions as a multimodal signal transducer of noxious stimuli in the mammalian somatosensory system [26]. Although chemical and thermal stimuli interact allosterically with TRPV1 through independent molecular mechanism, they can cross-sensitize each other to enhance channel activation. By characterizing the action mode of 1,4-dioxane at single-channel and whole-cell level, we unveiled its direct activation of TRPV1 and its potentiation effect on the activity of TRPV1 evoked by other stimulations. We also demonstrated that 1,4-dioxane significantly lowered the thermal threshold of TRPV1, which could explain the hyperalgesia behavior observed in vivo. The facilitatory effect of inflammatory factors on the activation of TRPV1 by 1,4-dioxane likely represents a positive feedback to amplify the pain sensation. These observations substantiate the theory of the synergistic sensitization among different stimuli on TRPV1 activation.

Functional and structural data have suggested the cytosolic S4-S5 linker as a gearbox in TRP channel gating [38]. Several residues within the S4-S5 linker of TRPV1 are critical to its function. For instance, numerous charged residues are important to the allosteric coupling between the voltage-, temperature-, 2-APB-, and capsaicin-dependent activation [39]. Structural analysis of TRPV1 protein has also identified the S4-S5 linker region as an important binding pocket for vanilloid and PI(4,5)P_2_ [40]. Furthermore, D. H. Kwan and colleagues found that M572 in the S4-S5 linker plays a critical role in the S6 gate opening and the mutation M572A nearly abolishes heat-dependent opening but with little effect on capsaicin response [41]. In the present study, we showed the substitution of M572 by V eliminated the 1,4-dioxane-evoked currents while retaining the capsaicin responses, thus further highlighting the importance of the S4-S5 linker in the channel gating. In addition, substitutions of M572 by A, L, and I with varied hydrophobic side-chain size in the S4-S5 linker ablated the activation of TRPV1 in varying degrees by 1,4-dioxane and its analogs without affecting the capsaicin response. However, the substitutions of M by F, W, R, E, or D resulted in nonfunctional channels. These results may arise from two possibilities. One is that methionine substituted into valine compromises the potential hydrophobic interaction between the sulfur atom and the aromatic ring-like compound like dioxanes [42], thus reducing the binding efficiency of 1,4-dioxane. It is remarkable that, despite the essential role of M572 in activation of TRPV1 by 1,4-dioxane, the actual binding sites of 1,4-dioxane on TRPV1 still requires further investigation, such as co-crystallization of this compound and channel protein. Another possibility is that M572 resides at a strategic position for channel gating, and its mutation might destroy the energetic transduction between 1,4-dioxane activation and gate opening. The present study identifies TRPV1 channel as a molecular target of 1,4-dioxane, which would help to understand its action on biological processes.

## Conclusions

We show that 1,4-dioxane targets in vivo the nociceptive capsaicin receptor TRPV1, whereby causing hyperalgesia in mouse model. With a mechanistic focus, we unveil that besides direct activation of TRPV1, 1,4-dioxane potentiates its agonistic and thermal activation. The effect of 1,4-dioxane is also upregulated by inflammatory factors, suggesting a positive feedback contributing to pain hypersensitivity. By site-directed mutagenesis, we further provide molecular insights into 1,4-dioxane-TRPV1 interaction. This study hence deepens our understanding on the pathological effect of 1,4-dioxane and its potential health concern.

## Methods

### Mutagenesis, cell culture, and transfection

The wild-type rat TRPV1, rat TRPV2, human TRPA1, mouse TRPA1, rat TRPM8, and mouse TRPV3 cDNAs were generously provided Dr. Feng Qin (State University of New York at Buffalo, Buffalo). All chimeras and site-directed mutagenesis were carried out using the overlap-extension polymerase chain reaction (PCR) method. Fragments of residues exchanged between TRPV1 and TRPV2 were as follows (TRPV1/TRPV2): P456-T476/P415-L436 for S1-S2 linker, Q498-S510/R458-S470 for S2-S3 linker, S532-M541/M492-L501 for S3-S4 linker, N551-L577/N511-L537 for S4-S5 linker, L585-L664/L547-L627 for S5-S6 linker, M1-F434/M1-F393 for N-terminus, and N687-K838/N650-P761 for C-terminus. All recombinant constructs and mutations were verified by DNA sequencing. HEK293 cells (ATCC, Manassas, VA, USA) were grown in Dulbecco’s modified Eagle’s medium (DMEM, Thermo Fisher Scientific, MA, USA) supplied with 4.5 mg/ml glucose, 10% heat-inactivated fetal bovine serum (FBS, Gibco, Thermo Fisher Scientific), 50 mg/ml penicillin, and 50 mg/ml streptomycin and were cultured at 37^o^C in a humidified incubator gassed with 5% CO_2_. Cells grown into ~80% confluence were transfected with the desired DNA constructs using either the standard calcium phosphate precipitation method or Lipofectamine 2000 (Invitrogen) following the protocol provided by the manufacturer. Transfected HEK293 cells were reseeded on 12 mm round glass coverslips coated by poly-l-lysine. Experiments took place usually 12–24 h after transfection.

### Mice

The *Trpv1*^-/-^ mice were generated by GemPharmatech Co., Ltd. through CRISPR/Cas9-mediated genome editing. In brief, the vectors encoding Cas9 and guide RNA (5'-GAGCCTAGGGCTAGGCA-3' and 5'-GCTACAGAGTGCAATTCCGGG-3') were in vitro transcribed into messenger RNA (mRNA) and gRNA followed by injection into the fertilized eggs that were transplanted into pseudopregnant mice. The targeted genome of F0 mice was amplified with PCR and sequenced, and the chimeras were crossed with wild-type C57BL/6 mice to obtain the *Trpv1*^+/-^ mice. The F1 *Trpv1*^+/-^ mice were further crossed with wild-type C57BL/6 mice for at least three generations. The age- and sex-matched *Trpv1*^+/+^ and *Trpv1*^-/-^ littermates were obtained by crossing the *Trpv1*^+/-^ mice and were randomly assigned to experimental groups throughout this study. The PCR primers for genotyping were as follows: *Trpv1*^*+/+*^ and *Trpv1*^*-/-*^ forward 5'-AGGGAGTCACTCAGGATGGCTTCT-3'; *Trpv1*^*+/+*^ reverse 5'-AGCCAGGTCAAGATTGGAA AAGG-3'; *Trpv1*^*-/-*^ reverse 5'- GCTGCCCTCTGACCTCAGCTACAC-3', with the expected product sizes of 375 bps and 558 bps for *Trpv1*^*-/-*^ and wild-type mice, respectively. All mice were housed in the specific pathogen-free animal facility at Wuhan University, and all animal experiments were in accordance with protocols (No. WDSKY0201804) approved by the Institutional Animal Care and Use Committee of Wuhan University.

### Hargreaves test

Behavioral studies were performed with 6- to 8-week-old wild-type or *Trpv1*^-/-^ adult C57 male mice. All tests were conducted during the light phase of the light/dark cycle by a trained observer blind to the genotype. Mice were habituated to the testing room for 60 min prior to all behavioral tests unless otherwise stated. The Hargreaves test was performed as described previously [32]. All behavioral experiments were conducted in a double-blind manner. Mouse was placed in a clear plexiglass cylinder on top of a temperature-controlled Plantar Test Instrument (Ugo Basile), which produces a high-intensity infrared light aimed at the plantar surface of the hind paw. The withdraw latency of thermal hyperalgesia was determined by the onset of paw lift and/or lifting, licking, and biting. Paw-withdrawal latency in response to heating was measured by a fixed infrared stimulus. Maximum stimulus duration was set at 20 s to prevent tissue damage. The animal was habituated in the plexiglass cylinder for 30 min. The right hind paws of mice were injected intraplantarly with 10 μl vehicle (normal saline). The left hind paws of mice were injected intraplantarly with 10 μl vehicle (normal saline supplemented with varying concentrations of 1, 4-dioxane as indicated). For measure of mechanical allodynia, a dynamic plantar aesthesiometer (von Frey apparatus, Ugo Basile, Milan, Italy) were used (stimulus rate of 1 g/s; cutoff value of 10 g). Each mouse was placed individually in clear Plexiglas chambers (8 × 8 × 12 cm) and acclimated for 30 min before testing. Mechanical threshold was measured at 30 min after injection. The mechanical threshold was averaged from up to three stimuli. Numbers of each group ≥ 6.

To develop the carrageenan-induced inflammation model, the mouse hind paw was injected with 20 μl 2% (w/v) carrageenan. In a half hour, the withdraw latency and the withdraw mechanical threshold were measured. Thereafter, 10 μl vehicle (normal saline supplemented with varying concentrations of 1, 4-dioxane as indicated) was injected into the same hind paws post carrageenan injection. The withdraw latency and the withdraw mechanical threshold were determined again 30 min later.

### Paw edema test

Edema was induced by intraplantar injection of 10 μl of 1,4-dixoane freshly prepared in vehicle (normal saline supplemented with 5% 1, 4-dioxane) into the left-hind paws of *Trpv1*^+/+^ and *Trpv1*^-/-^ mice, respectively. Paw volumes were measured just before and 2 h post injection of 1,4-dixoane using a plethysmometer (IITC). The increase in percentage of paw volume was calculated based on the volume difference between the normal and abnormal paws (with or without injection of 1, 4-dioxane). The following equation was used: paw edema ratio (%) = (paw volume after injection of 1, 4-dioxane—paw volume after injection of saline)/paw volume after injection of saline × 100%.

### Preparation of dorsal root ganglia (DRG) neurons and trigeminal ganglia (TG)

Sensory neurons were dissociated from dorsal root ganglia (DRG) or trigeminal ganglia (TG) of mice. Primary culture of DRG neurons and TG neurons were established following enzymatically and mechanically dissociation of the ganglia as described before with minor modification [43, 44]. Briefly, 6- to 8-week-old wild-type or *Trpv1*^-/-^ C57 BL/6 mice were used. Mice were deeply anesthetized and then decapitated. DRGs together with the dorsal-ventral roots and attached spinal nerves were taken out from the spinal column. After removing the attached nerves and surrounding connective tissues, about 10–14 DRGs from the thoracic and lumbar segments of spinal cords were rapidly dissected and cleaned in Ca^2+^/Mg^2+^-free Hank’s balanced salt solution (HBSS). Ganglia were dissociated by enzymatic treatment with collagenase type IA (1 mg/ml), trypsin (0.4 mg/ml), and DNase I (0.1 mg/ml) and incubated at 37^o^C for 40 min. After dissection of the paired TG, they were washed in phosphate-buffered saline (PBS), minced and gathered in minimal essential medium (MEM, Invitrogen) containing collagenase type IA (1 mg/ml), and incubated at 37^o^C for 45 min. During digestion, gentle mechanical trituration was performed every 10 min through fire-polished glass pipettes until solution became cloudy. The resulting suspension of single cells was collected by centrifuge. After three washes in DMEM/F12 medium containing 10% FBS, 50 units/ml penicillin, and 50 μg/ml streptomycin, cells were dispersed by gentle titration, plated on glass coverslips coated with poly-l-lysine, and cultured at 37^o^C in a humidified incubator with 5% CO_2_. Patch-clamp recordings were carried out 12–24 h after plating. TRPV1(WT), TRPV1(M572V), or empty vector together with the expression marker GFP was re-introduced into *Trpv1*^*-/-*^ DRGs by electroporation using Neon transfection system (Life Technologies) as described [45].

### Ca^2+^ imaging

Cells on coverslips were loaded with 5 μM Fluo-4 AM (Beyotime Bio-Tech Co., Ltd.) for 40 min at 37^o^C in the original culture room. The cells were then washed three times with an incubation buffer containing (in mM) 140 NaCl, 5 KCl, 2 MgCl_2_, 10 HEPES, 10 glucose, and 2 CaCl_2_ (pH 7.4). Cells were incubated in incubation buffer for 30 min at 37^o^C to allow deesterification of intracellular AM esters. Calcium imaging was performed on an inverted epifluorescence microscope (Olympus IX 73) equipped with a complete illumination system (Lambda XL, Sutter Instruments). Fluorescent images were acquired using a cool CCD camera (CoolSNAP ES2, Teledyne Photometrics) controlled by Micro-Manager 1.4 (Vale lab, UCSF) using a public 1394 digital camera driver (Carnegie Mellon University). Images at excitation of 448–492 nm and emission of 512–630 nm were taken in a continuous time-based mode. After baseline images were taken, 1,4-dioxane was added. High KCl (60 mM) was applied in the end of each experiment to ascertain neuronal viability. More than 90% neurons were responsive to 60 mM KCl stimulation and were included in the analysis.

### Electrophysiological recordings

Conventional whole-cell and excised outside-out patch-clamp recording methods were used. For the recombinant expressing system, EGFP fluorescence was used as a marker for gene expression. Patch-clamp recordings were voltage clamped using an EPC10 amplifier (HEKA, Lambrecht, Germany). Voltage commands were made from the Patchmaster program. For a subset of recordings, currents were amplified using an Axopatch 200B amplifier (Molecular Devices, Sunnyvale, CA) and recorded through a BNC-2090/MIO acquisition system (National Instruments, Austin, TX) using QStudio developed by Dr. Feng Qin at State University of New York at Buffalo. Recording pipettes were pulled from borosilicate glass capillaries (World Precision Instruments), and fire-polished to a resistance between 2 and 4 MΩ for whole-cell recordings, and 6–10 MΩ for single-channel recordings when filled with internal solution. Whole-cell recordings were typically sampled at 5 kHz and filtered at 1 kHz, and single-channel recordings were sampled at 25 kHz and filtered at 10 kHz.

Bath saline for whole-cell recording in HEK293 cells consisted of (in mM): 140 NaCl, 5 KCl, 5 EGTA, and 10 HEPES, pH 7.4 adjusted with NaOH. MES [2-(*N*-morpholino)ethanesulfonic acid] was used as the pH buffer when pH<6.5 and HEPES was used for pH 7.0–7.4. Electrodes were filled with (in mM): 140 CsCl, 5 EGTA, 10 HEPES, and pH 7.4 adjusted with CsOH. The bath and pipette solutions for single-channel recording were symmetrical and contained 140 NaCl, 5 KCl, 5 EGTA, and 10 HEPES, pH 7.4 (adjusted with NaOH). For recording DRG and TG neurons, the bath solution contained (in mM): 140 NaCl, 5 KCl, 2 MgCl_2_, 2 CaCl_2_, 10 glucose, 10 HEPES, and pH 7.4 adjusted with NaOH and the pipette solution contained (in mM): 140 CsCl, 5 NaCl, 5 EGTA, and 10 HEPES, pH 7.3 adjusted with CsOH. Exchange of external solutions was performed using a gravity-driven local perfusion system. As determined by the conductance tests, the solution around a patch under study was fully controlled by the application of a solution with a flow rate of 100 μl/min or greater. All pharmacological experiments met this criterion. For recordings under low pH conditions, the solution also contained 50 μM amiloride to inhibit native acid sensing ion channels. Capsaicin and capsazepine were dissolved in pure ethanol to make a stock solution. All the stocks were diluted in the bath solution to the desired concentrations right before the experiment. The final concentrations of ethanol did not exceed 0.3%, which had no effect on the currents. Unless otherwise noted, all chemicals were purchased from Sigma (Millipore Sigma, St. Louis, MO). The suppliers, catalog numbers and storage methods of some major chemical reagents are shown in Table 1. All patch-clamp recordings were performed at room temperature (RT, 22–24^o^C) except for temperature stimulation (see below).

**Table 1:** Specification of the major chemical reagents

Chemicals	Supplier	Purity	Identifiers	Storage	Solvent
1,4-Dioxane	Sigma-Aldrich	≥99.8%, anhydrous	Cat.#: 296309	RT	DMSO
1,3-Dioxane	Sigma-Aldrich	97%	Cat.#: 283061	RT	DMSO
Tetrahydrofuran	Sigma-Aldrich	≥99.9%, anhydrous	Cat.#: 401757	RT	DMSO
1,3,5-Trioxane	Sigma-Aldrich	≥99%	Cat.#: T81108	RT	DMSO
Capsaicin	MCE	98.86%	Cat.#: HY10448	-20 ^o^C	Anhydrous ethanol
2-APB	Sigma-Aldrich	97%	Cat.#: D9754	-20 ^o^C	DMSO
Ruthenium Red	Sigma-Aldrich	Technical grade	Cat.#: R2751	RT	Deionized water
AITC	Sigma-Aldrich	95%	Cat.#: 377430	4 ^o^C	DMSO
Menthol	Sigma-Aldrich	≥98%	Cat.#: M2547	4 ^o^C	DMSO
Pregnenolone	TargetMol	≥99.5%	Cat.#: T0851	-20 ^o^C	DMSO

### Ultrafast temperature jump achievement

A single-emitter infrared laser diode (1470 nm) was designed to produce temperature jumps, as previously described [46]. A multimode fiber with a core diameter of 100 μm was used to transmit the launched laser beam. The other end of the fiber exposed the fiber core was placed close to the cell of interest where the perfusion pipette is usually located. The laser diode was driven by a pulsed quasi-CW current power supply (Stone Laser, Beijing, China), and pulsing of the controller was controlled from a computer through BNC-2090/MIO data acquisition card, which was also responsible for patch-clamp recordings. A blue laser line (460 nm) was coupled into the same fiber to aid alignment. Constant temperature steps were generated by irradiating the tip of an open pipette filled with the bath solution and the current through the electrode was used as a readout for feedback control. The laser diode was first powered on for a brief duration to reach the set temperature and was then modulated to achieve a constant pipette current. The profile of the modulation pulses was stored and subsequently played back to apply the temperature jump to the cell of interest. Temperature was calibrated offline from the electrode current based on the temperature dependence of electrolyte conductivity. The threshold temperature for heat activation of TRPV1 was determined as the temperature at which the slow inward current was elicited.

### Statistics and reproducibility

Data were processed with Qstudio developed by Dr. Feng Qin at State University of New York at Buffalo, Clampfit (Molecular Devices, Sunnyvale, CA), IGOR (Wavemetrics, Lake Oswego, OR, USA), SigmaPlot (SPSS Science, Chicago, IL, USA), and OriginPro (OriginLab Corporation, MA, USA). For concentration response analysis, the modified Hill equation was used: *Y* = A1 + (A2–A1)/[1 + 10 ^ (logEC_50_ – X) * n_*H*_], in which EC_50_ is the half maximal effective concentration, and n_*H*_ is the Hill coefficient. Experiments were performed in two or more separate batches of DRG, TG, and HEK293 cells to confirm reproducibility. Unless stated otherwise, the summary data are presented as mean ± standard error (s.e.m.), from a population of cells (*n*) with statistical significance assessed by Student’s *t* test for one or two group comparison and one-way analysis of variance (ANOVA) tests for multiple group comparisons. Significant difference is indicated by a *p* value less than 0.05 (*p < 0.05, **p < 0.01, *** *p* < 0.001).

## Supplementary Information

**Additional File 1: Figures S1-S5. Fig. S1.** Effects of 1,4-dioxane on variable TRP channels. **Fig. S2.** Generation of Trpv1 KO mice. **Fig. S3.** Amplitude of TRPV1 single-channel currents elicited by different conditions. **Fig. S4.** Inhibitory effect of ruthenium red (RR) on TRPV1 currents. **Fig. S5.** Reintroducing of TRPV1(M572V) into Trpv1-/- DRG neurons only rescues the responses to capsaicin but not 1,4-dioxane.

## Data Availability

All major datasets supporting the conclusions of this article are available in Dryad, doi:10.5061/dryad.x3ffbg7h1 [47].
