# Supplementary material for: The industrial solvent 1,4-dioxane causes hyperalgesia by targeting capsaicin receptor TRPV1
Source: BMC Biol. 2022 Jan 7;20:10. doi: 10.1186/s12915-021-01211-0 (PMC8742357; doi:10.1186/s12915-021-01211-0)
Supplement: Supplementary file 1 — Additional File 1: Figures S1-S5. Fig. S1. Effects of 1,4-dioxane on variable TRP channels. Fig. S2. Generation of Trpv1 KO mice. Fig. S3. Amplitude of TRPV1 single-channel currents elicited by different conditions. Fig. S4. Inhibitory effect of ruthenium red (RR) on TRPV1 currents. Fig. S5. Reintroducing of TRPV1(M572V) into Trpv1-/- DRG neurons only rescues the responses to capsaicin but not 1,4-dioxane. [file 12915_2021_1211_MOESM1_ESM.docx]

**Supplementary Information**

**The industrial solvent 1,4-Dioxane causes hyperalgesia by targeting capsaicin receptor TRPV1**

Xiaoyi Mo^1,3^, Qiang Liu^1,3^, Luna Gao^1,3^, Chang Xie^1,3^, Xin Wei^1^, Peiyuan Pang^1^, Quan Tian^1^, Yue Gao^1^, Youjing Zhang^1^, Yuanyuan Wang^1^, Tianchen Xiong^1^, Bo Zhong^1^, Dongdong Li^2^, Jing Yao^1🖂^

^1^ State Key Laboratory of Virology, College of Life Sciences, Department of Anesthesiology, Zhongnan Hospital of Wuhan University, Frontier Science Center for Immunology and Metabolism, Wuhan University, Wuhan, Hubei 430072, China

^2^ Sorbonne Université, Institute of Biology Paris Seine, Neuroscience Paris Seine, CNRS UMR8246, INSERM U1130, UPMC UM119, Paris 75005, France

^3^ These authors contributed equally to this work.

**Running title:** Gating of TRPV1 channels by 1, 4-dioxane

🖂 Address correspondence to:

Dr. Jing Yao

State Key Laboratory of Virology,

College of Life Sciences,

Department of Anesthesiology, Zhongnan Hospital of Wuhan University,

Frontier Science Center for Immunology and Metabolism,

Wuhan University,

Wuhan, Hubei 430072, China

Phone: 86-27-68752148

Email: [jyao@whu.edu.cn](mailto:jyao@whu.edu.cn)

**Supplementary Figure 1**

**
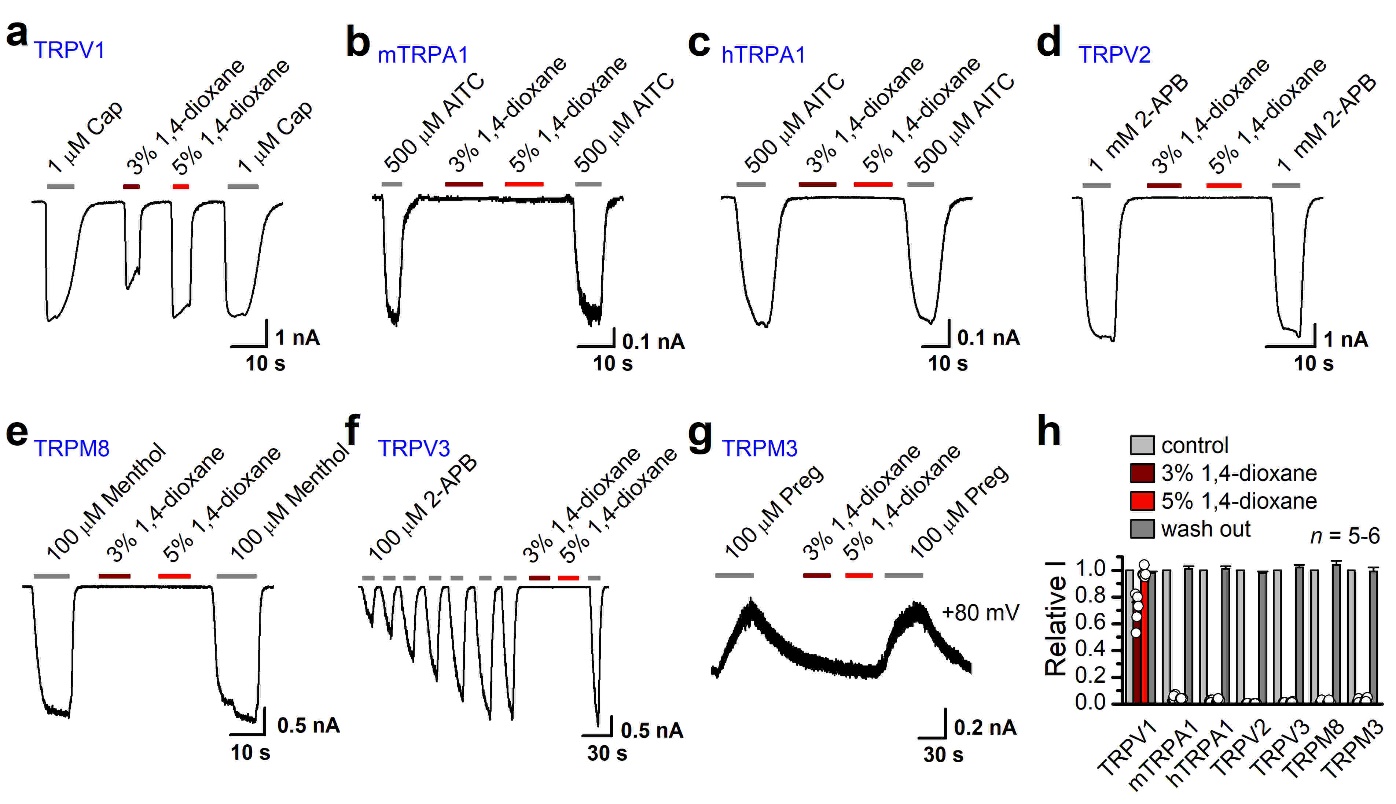
**

**Figure S1. Effects of 1,4-dioxane on variable TRP channels.**

(**a-g**) Representative current traces from whole-cell voltage-clamp recordings show different effects of 1,4-dioxane (3% or 5%) on TRPV1(**a**), mouse TRPA1(**b**), human TRPA1(**c**), TRPV2(**d**), TRPM8(**e**), TRPV3(**f**), or TRPM3(**g**) channels, which were however efficiently activated by their own agonists Cap (Capsaicin), AITC (Allyl Isothiocyanate), 2-APB, Menthol and Preg (Pregnenolone). Bars represent duration of drug application. Except the holding potential for TRPM3 channel current which was +80 mV, the holding potential for other channels was -60 mV. (**h**) Summary of relative currents. There were no measurable currents evoked by 3% or 5% 1,4-dioxane in cells that transiently expressed TRPA1, TRPV2, TRPV3, TRPM8 or TRPM3 channels (*n* = 5-6).

**Supplementary Figure 2**

**
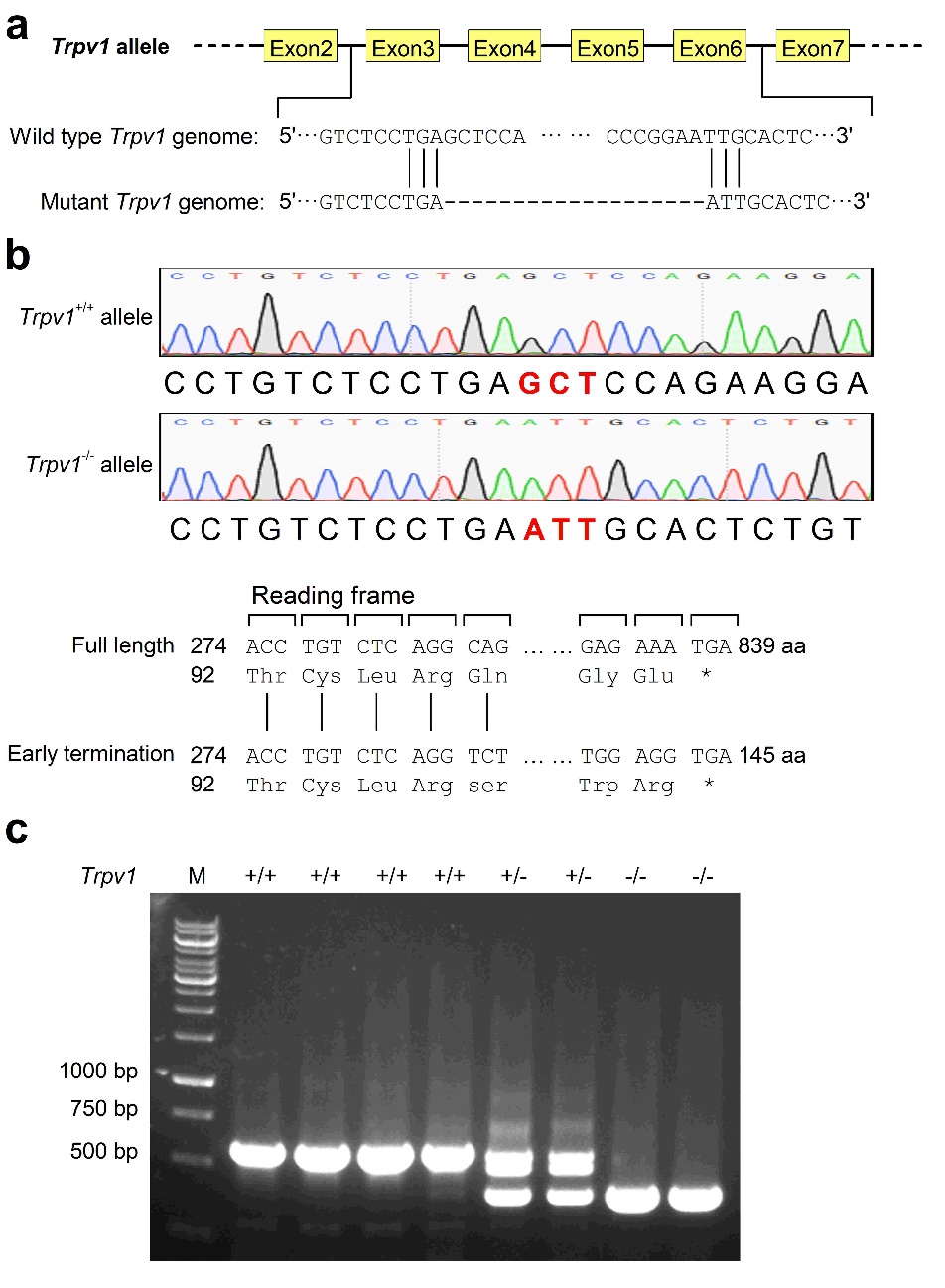
**

**Figure S2. Generation of Trpv1 KO mice.**

(**a**) CRIPSR/Cas9-mediated genome editing of the *Trpv1* gene locus. (**b**) Gene sequence and reading frame of *Trpv1*^+/+^ and *Trpv1*^-/-^ mice. (**c**) PCR analysis of genomic DNA obtained from *Trpv1*^+/+^, *Trpv1*^+/-^, and *Trpv1*^-/-^ mice.

**Supplementary Figure 3**

**
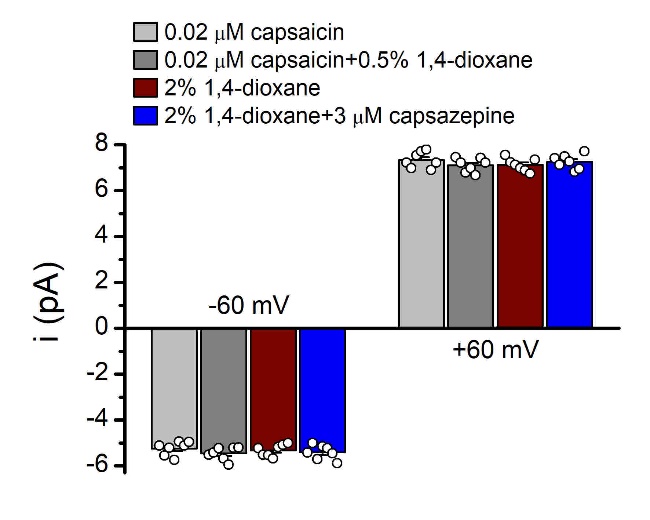
**

**Figure S3. Amplitude of TRPV1 single-channel currents elicited by different conditions.**

No significant difference in averaged amplitude of TRPV1 single-channel currents recorded from outside-out patches of TRPV1-expressing HEK293 cells as shown in Figure 2h.

**Supplementary Figure 4**

**
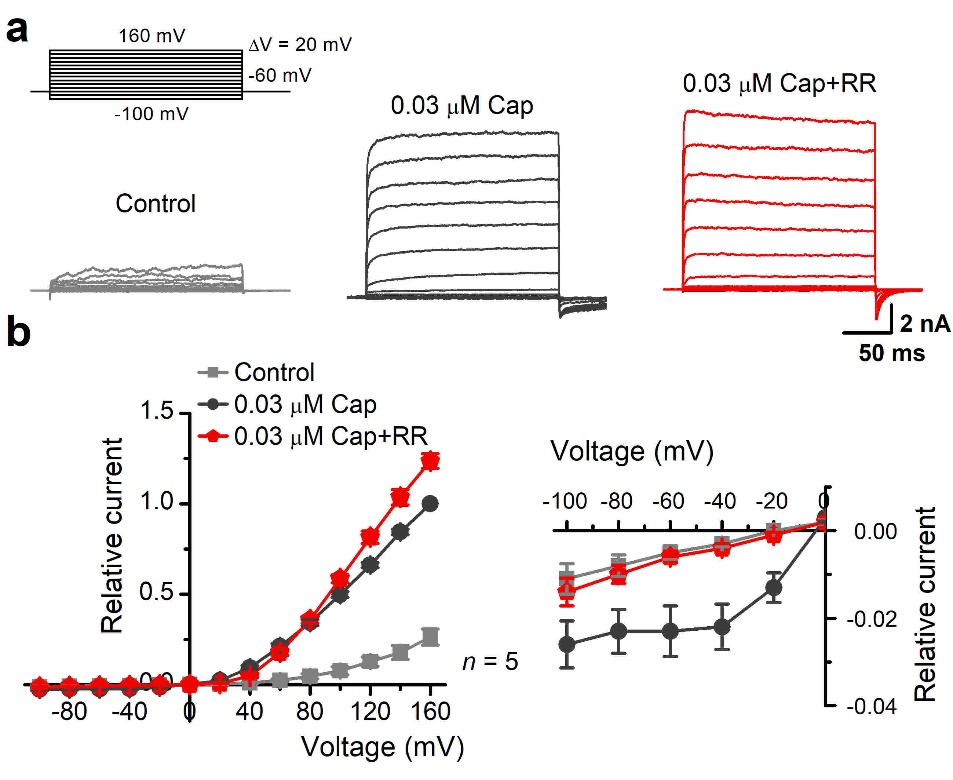
**

**Figure S4. Inhibitory effect of ruthenium red (RR) on TRPV1 currents.**

(**a**) Representative whole-cell currents evoked by voltage steps (inset) without or with 0.03 μM capsaicin (Cap) in the absence and presence of 10 μM RR in HEK 293 cells expressed TRPV1 channel. Currents were elicited with 200 ms test pulses ranging from –100 mV to +160 mV in 20 mV increments from holding potential of -60 mV within the same cell. (**b**) Normalized current-voltage relations for data in (**a**). Current amplitudes were normalized to the maximum responses at +160 mV in the presence of 0.03 μM capsaicin. Each point represents mean values (± SEM) from five independent cells. The inhibitory effect of RR on TRPV1 currents at negative holding potentials are magnified and displayed on the right.

**Supplementary Figure 5**

**
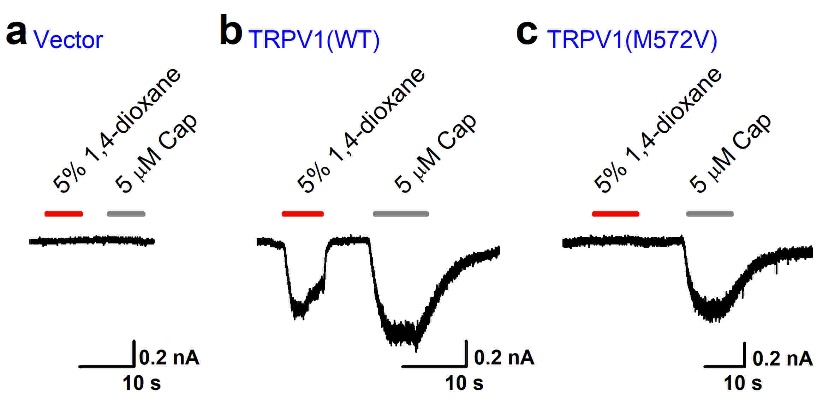
**

**Figure S5. Reintroducing of TRPV1(M572V) into *Trpv1^-/-^* DRG neurons only rescues the responses to capsaicin but not 1,4-dioxane.**

(**a**-**c**) Representative whole-cell recordings were performed after 36-48 h after re-expression of empty vector (**a**), TRPV1(WT) (**b**) or TRPV1(M572) (**c**) in *Trpv1^-/-^* DRG neurons by electroporation. The cells were consecutively exposed to 1,4-dioxane (5%) and capsaicin (Cap, 5 μM), as indicated. Holding potential was -60 mV.
